# Effects of maternal and paternal smoking on offspring cardiometabolic risk factors in adulthood: a multi-method intergenerational Mendelian randomization study

**DOI:** 10.1186/s12916-026-05065-7

**Published:** 2026-08-05

**Authors:** Grace M. Power, Tom A. Bond, Laxmi Bhatta, Bjørn Olav Åsvold, Ben Brumpton, Giulio Centorame, Gibran Hemani, Deborah A. Lawlor, Geng Wang, Nicole Warrington, David M. Evans, Gunn-Helen Moen, George Davey Smith

**Affiliations:** 1 MRC Integrative Epidemiology Unit at the University of Bristol, Bristol, UK; 2 Population Health Sciences, Bristol Medical School, University of Bristol, Bristol, UK; 3 Institute for Molecular Bioscience, University of Queensland, Brisbane, QLD Australia; 4 Frazer Institute, University of Queensland, Woolloongabba, QLD Australia; 5 Department of Epidemiology and Biostatistics, Imperial College London, London, UK; 6 HUNT Center for Molecular and Clinical Epidemiology, Department of Public Health and Nursing, NTNU Norwegian University of Science and Technology, Trondheim, Norway; 7 HUNT Research Centre, Department of Public Health and Nursing, NTNU Norwegian University of Science and Technology, Levanger, Norway; 8 Department of Endocrinology, Clinic of Medicine, St. Olavs Hospital, Trondheim University Hospital, Trondheim, 7030 Norway; 9 Clinic of Medicine, St. Olavs Hospital, Trondheim University Hospital, Trondheim, Norway; 10 Institute of Clinical Medicine, Faculty of Medicine, University of Oslo, Oslo, Norway; 11 NIHR Bristol Biomedical Research Centre Bristol, University Hospitals Bristol and Weston NHS Foundation Trust, University of Bristol, Bristol, UK

**Keywords:** Intergenerational epidemiology, Mendelian randomization, Parental smoking, Long-term offspring health, Cardiometabolic risk

## Abstract

**Background:**

Parental smoking has been linked to several adverse offspring cardiometabolic outcomes; however, evidence is conflicting regarding the causal and long-term nature of these associations.

**Methods:**

We investigated the effects of maternal and paternal smoking, capturing exposure before, during, and after pregnancy, on eleven offspring cardiometabolic risk factors related to body composition, blood pressure, glucose, and lipid levels in adulthood. We applied a multi-method intergenerational Mendelian randomization (MR) framework, combining two-sample MR (outcome GWAS, n = up to 564,160) and one-sample MR analyses (n = up to 17,484 genotyped mother-father-offspring trios with offspring cardiometabolic risk factors) from the HUNT cohort, Norway, and the UK Biobank and ALSPAC cohorts in the United Kingdom. Smoking behaviour was instrumented using genome-wide significant variants for smoking initiation and heaviness from large genome-wide association studies (2019 and 2022), with additional analyses of the *CHRNA5* variant rs16969968.

**Results:**

Using the two-sample MR approach, we found an average change in adult offspring waist-hip ratio (WHR) per one standard deviation (SD) increase in maternal cigarettes smoked per day of 0.25 SD (95% CI: 0.13, 0.38; 2019 GWAS), 0.19 SD (95% CI: 0.08, 0.31; 2022 GWAS) and 0.27 SD (95% CI: 0.07, 0.47; *CHRNA5* rs16969968). We also found tentative evidence of comparable effects on offspring body mass index and C-reactive protein. One-sample MR analyses using *CHRNA5* rs16969968, restricted to maternal ever-smokers, provided supporting evidence consistent with the primary findings for maternal smoking heaviness on offspring WHR. We found little evidence that maternal smoking initiation affected WHR, and little evidence that maternal smoking heaviness affected the remaining cardiometabolic risk factors; paternal smoking showed no clear effect on any outcome. Results from sensitivity analyses were consistent with these main findings.

**Conclusions:**

Adult offspring of mothers with a propensity to heavier smoking exhibited greater central adiposity, a phenotype associated with increased cardiometabolic risk. These findings are consistent with a causal influence of maternal smoking heaviness across the preconception, perinatal, and postnatal periods on later-life offspring body fat distribution. Further triangulation using alternative causal approaches is warranted.

## Background

Cardiovascular diseases (CVDs) and related cardiometabolic disorders are among the leading causes of morbidity and mortality worldwide [1]. The Developmental Origins of Health and Disease (DOHaD) framework postulates that the development of adult cardiovascular and metabolic disease is influenced by the environment in the preconception, fertilization, embryonic, fetal, and neonatal periods [2, 3]. Environmental insults in early life are hypothesised to impede development, resulting in the permanent ‘programming’ of endocrine and metabolic function [4, 5], subsequently heightening susceptibility to cardiometabolic disease in adulthood [2, 6–9].

Smoking by mothers during pregnancy is a well-established modifiable risk factor for several adverse perinatal health outcomes [10–12]. Whilst attention has largely focused on the effects of maternal smoking on conception, pregnancy, and fetal and child health, there have been concerns that perinatal exposure to smoking may play an important role in the increased risk of cardiometabolic disorders in offspring in their later life [11, 13]. Previous conventional epidemiological analyses have shown that parental smoking is associated with increased incidence of later life obesity and related comorbidities. For example, a meta-analysis comprising 19 multivariable regression studies (n = 24,201 to 308,981) found that maternal prenatal smoking was associated with higher risks of overweight, obesity, and gestational diabetes mellitus in adult offspring, but not type 2 diabetes, hypertension, waist circumference, or total cholesterol [14]. In addition, an individual patient data meta-analysis which included 26 studies of mother-child pairs (n = 238,340) indicated a positive linear dose-response relationship between the number of cigarettes smoked by mothers during pregnancy and offspring overweight in childhood and adolescence for up to 15 cigarettes per day [15]. An association has also been identified between paternal smoking during preadolescence and increased body mass index (BMI), fat mass, and waist circumference in male offspring, with no corresponding effect observed in female offspring [16]. However, a larger study conducted in the Trøndelag Health Study (HUNT), designed to reproduce these earlier findings, found little evidence that exposure to parental smoke in preadolescence was associated with offspring BMI at a mean age of 29.1 years [17]. Epigenetic studies have suggested that maternal and/or paternal smoking may affect deoxyribonucleic acid (DNA) methylation in the offspring, including robust evidence for persistent methylation changes following maternal smoking in pregnancy, providing a plausible mechanism by which parental smoking could increase offspring obesity risk [18, 19].

Mendelian randomization (MR) studies in the general population support a causal effect of smoking behaviours on cardiometabolic traits and cardiovascular disease [20–22]. The evidence for a causal effect of parental smoking on offspring cardiometabolic risk is contradictory, however. A proxy gene-by-environment MR study found little evidence for a causal effect of maternal smoking on 12 long-term offspring outcomes, including BMI and blood pressure, although the authors acknowledged a lack of power to detect such effects [23]. In addition, studies employing a sibling comparison design, which contrasts siblings with discordant exposure to maternal smoking during pregnancy (with the aim of controlling for confounders that are shared by siblings), concluded that the observed associations between maternal smoking during pregnancy and offspring obesity were more likely to be confounded by shared familial characteristics than reflect a causal intrauterine mechanism [24, 25]. Earlier paternal negative control studies suggested that maternal smoking in pregnancy showed larger associations with childhood overweight and obesity than paternal smoking in mutually adjusted models, which was interpreted as being more consistent with a causal intrauterine effect [26]. This study design assumes that if a causal intrauterine effect of the maternal exposure on the offspring is present, the association of the maternal exposure with the outcome should be larger than the association of the equivalent paternal exposure [27]. However, evidence from an individual participant data meta-analysis of 229,158 families shows that maternal and paternal smoking have similar associations with childhood overweight, a pattern more consistent with shared familial confounding than with a specific intrauterine effect [28].

Plausible explanations for the identification of an association between maternal or paternal smoking and cardiometabolic outcomes in adult offspring include: (i) consequences from noxious chemicals found in cigarette smoke, impacting the oocyte or sperm, (ii) adverse intrauterine effects from associated chemical insults, (iii) postnatal exposure to parental smoking for the offspring, or (iv) potential confounding bias. In conventional epidemiological settings, biases induced by confounding pose challenges when inferring causality. In intergenerational settings, parents and offspring share social, environmental, and behavioural factors, introducing distinctive sources of confounding [29, 30]. Associations identified could be due to environmental confounding or to shared familial and genetic effects [31].

Mendelian randomization (MR) is a technique that exploits the random distribution of genetic variants from parents to offspring, independent of the influence of other traits. Provided certain assumptions are met, MR reduces susceptibility to confounding factors, including confounding by undiagnosed existing disease (reverse causation) [32, 33]. In intergenerational analyses, MR approaches that account for direct genetic transmission from parent to offspring can help mitigate bias arising from shared familial structure [34]. Recent developments and applications in intergenerational genome-wide association studies (GWAS) and Mendelian randomization (MR) methodology [34–37] enable estimation of the indirect effects of parental exposures on offspring outcomes in adulthood by accounting for the direct fetal genotype contribution [6].

We aimed to use intergenerational MR to test the hypothesis that maternal and paternal smoking before, during, and after pregnancy causes a more adverse offspring cardiometabolic risk factor profile in adulthood.

## Methods

This study adheres to the Strengthening the Reporting of Observational Studies in Epidemiology Using Mendelian Randomisation (STROBE-MR) guidelines [38, 39] (Additional file 1).

### Data sources and instruments

#### Inclusion criteria and participating cohorts

**Fig. 1:**
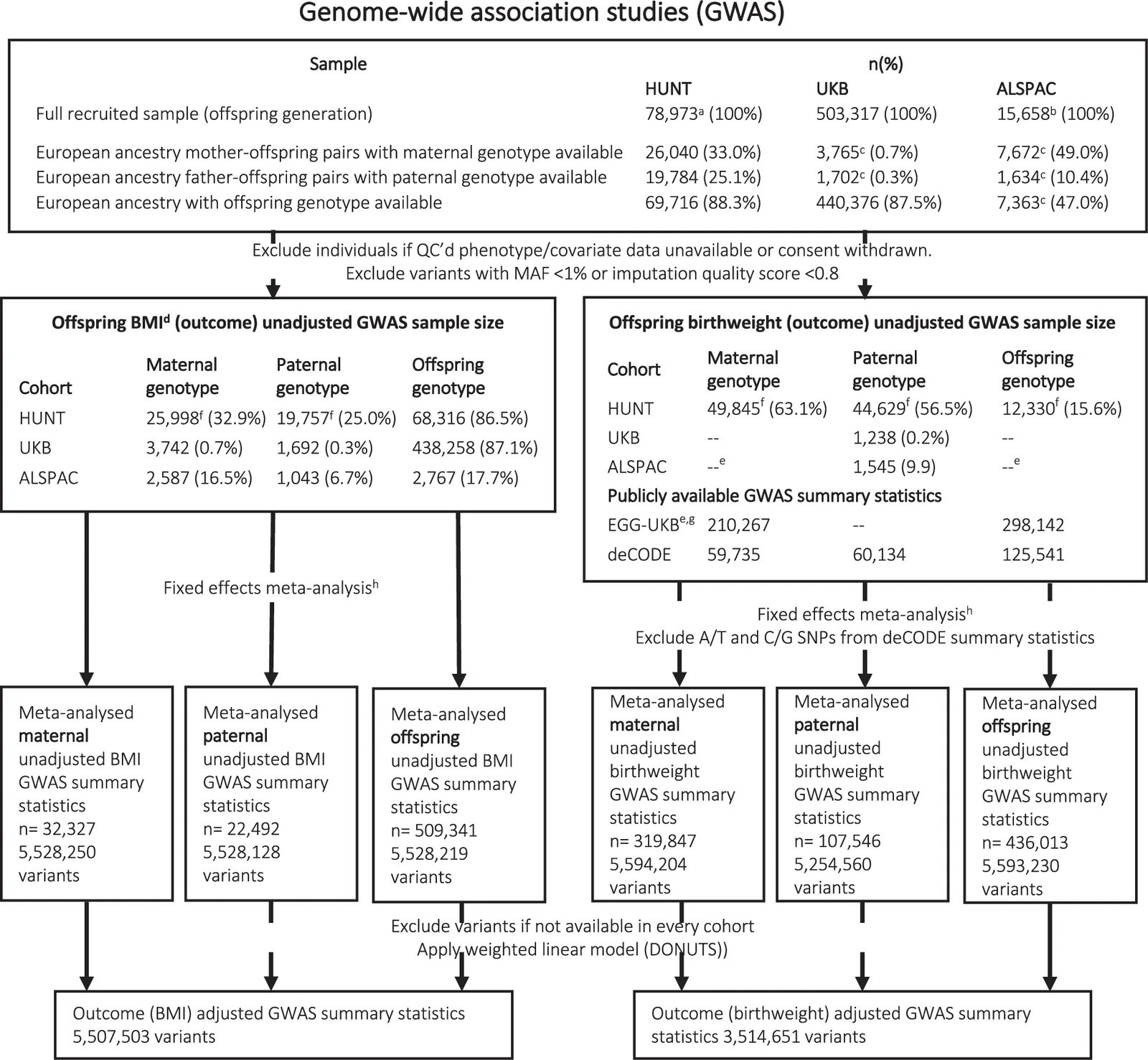
Study design overview and flowchart for instruments used in two-sample MR (GWAS) (Figure adapted from Bond and Bhatta 2025 [35, 36]). BMI: body mass index, BW: birthweight, HUNT: Trøndelag Health Study, UKB: UK Biobank, ALSPAC: Avon Longitudinal Study of Parents and Children, EGG: Early Growth Genetics Consortium, GIANT: Genetic Investigation of Anthropometric Traits Consortium, GWAS: genome-wide association study, QC: quality control, MAF: minor allele frequency. a: total number of participants in the HUNT2 and HUNT3 study waves, b: total number of fetuses included in ALSPAC (including participants recruited after 7 years of age), c: only unrelated individuals within the parent and offspring generations were included in the designated samples, d: sample sizes were similar for the other adult outcomes, e: ALSPAC data were included in EGG, f: in HUNT, the parent-offspring samples included siblings in the offspring generation, and for the birthweight analyses parent-offspring pairs were identified via a birth registry instead of via genetic data, resulting in larger samples for parental GWAS and a smaller sample for offspring GWAS compared with adult outcomes, g: we used publicly available summary statistics from the meta-analysis of EGG, UKB, and deCODE carried out by Juliusdottir et al. [110], h: prior to meta-analysis, A/T and C/G SNPs were removed when comparison of their allele frequency to the HRC or 1000 Genomes Project reference panel suggested harmonisation errors

We analysed three large population-based prospective cohorts that had genome-wide data available in mothers, fathers, and offspring and cardiometabolic risk factors measured in adult offspring: the Trøndelag Health Study (HUNT), the UK Biobank (UKB), and the Avon Longitudinal Study of Parents and Children (ALSPAC). The selection of study participants for our main two-sample MR analysis is presented in Fig. 1. Study participant selection for one-sample MR is presented in Additional file 1: Figure S1.

##### HUNT

HUNT is a cohort study of the adult population in Trøndelag County, Norway, and comprises ~230,000 individuals aged ≥20 years [40, 41]. Individuals were recruited in four surveys from 1984–2019, of which the HUNT2 and HUNT3 surveys were included in the present study. Invitee participation rates were 69% and 54%, respectively. Data on perinatal outcomes are available via linkage to the Medical Birth Registry of Norway (MBRN) [42] for individuals born after 1967. A total of 69,716 European ancestry offspring were available in HUNT, with genotype data available, of which 26,040 had maternal genotype data available and 19,784 had paternal genotype data available [6, 43, 44]. Parent-offspring pairs and trios were identified using kinship analysis implemented in the KING software package [45].

##### UKB

UKB is a cohort of 503,317 adult volunteers, recruited from across the UK aged 40–69 years between 2006 and 2010 [46, 47]. Parent-offspring pairs and trios were identified using kinship analysis implemented in the KING software package [45].

##### ALSPAC

ALSPAC is a birth cohort which enrolled 14,541 pregnant women living in Avon, England with expected dates of delivery between April 1, 1991, and December 31, 1992 (the estimated recruitment rate of those eligible was 80%) [48, 49]. Further enrolments after 1998 resulted in a baseline sample of 14,901 children alive at one year of age. We used data collected during pregnancy/birth and the 24-year follow-up. Some study data were collected and managed using Research Electronic Data Capture (REDCap) electronic data capture tools hosted at the University of Bristol [50]. Please note that the study website contains details of all the data that is available through a fully searchable data dictionary and variable search tool: http://www.bristol.ac.uk/alspac/researchers/our-data/. Parent-offspring pairs and trios were identified by matching on study ID variables.

#### Offspring cardiometabolic risk factors and birthweight

Standard protocols were used to assess the eleven offspring cardiometabolic outcomes in adulthood (body mass index (BMI), waist-hip ratio (WHR), systolic (SBP) and diastolic (DBP) blood pressure, blood glucose, glycated haemoglobin (HbA1c), total cholesterol (TC), high-density lipoprotein cholesterol (HDL-C), low-density lipoprotein cholesterol (LDL-C), triglycerides and high sensitivity C-reactive protein (CRP)) [35, 36]. Outcomes were derived from adult clinic assessments in each cohort. In HUNT, where measurements were available from both HUNT2 (1995–1997) and HUNT3 (2006–2008), the most recent available value for everyone was used. In UK Biobank, outcomes were obtained from the baseline assessment visit (2006–2010); repeat assessment visits were not used. In ALSPAC, outcomes were obtained from the 24-year follow-up clinic. For all cohorts, where more than one blood pressure measurement was recorded at a given visit, the mean value was used, as described previously [36].

For one-sample MR analyses, CRP was only available in ALSPAC and UKB and HbA1c was only available in UKB. Blood pressure and lipid measurements were corrected for antihypertensive and lipid-lowering medication use as described previously [35, 36]. Briefly, blood pressure values for participants in HUNT and UKB who reported taking antihypertensive medication were corrected by adding 15 mmHg and 10 mmHg to the raw SBP and DBP values, respectively. Lipid values were corrected for participants reporting taking lipid-lowering medication in UKB by dividing the raw LDL cholesterol values by 0.7 and the raw total cholesterol and triglyceride values by 0.8 [35, 36]. Biochemical values were measured in fasting blood samples in ALSPAC and non-fasting in HUNT and UKB. Offspring birthweight was employed as a positive control outcome in this analysis. A priori, we expected to see a negative causal effect of smoking during pregnancy on birthweight [51–53].

Birthweight was extracted from the Medical Birth Registry of Norway (MBRN) in HUNT, measured by trained research assistants or abstracted from the birth record/notification in ALSPAC, and retrospectively self-reported in UKB. CRP was strongly right-skewed and therefore was natural log-transformed prior to GWAS.

#### Genotyping, quality control and imputation in each cohort

Genotyping, quality control (QC) and imputation in each cohort are described in detail in Additional file 1 and elsewhere [6, 47, 54–62]. To summarise, participants were genotyped using genome-wide single nucleotide polymorphism (SNP) arrays, followed by imputation of genome-wide variants. HUNT participants were imputed using a customised Haplotype Reference Consortium (HRC) panel including 2,201 Norwegian whole genome sequences. ALSPAC mothers and offspring were imputed to the HRC panel [43, 61], and ALSPAC fathers to the 1000 Genomes phase 1 panel [63]. UKB participants were imputed to a merged HRC/UK10K panel [47]. Imputed variants passing cohort-specific QC criteria, including minor allele frequency (MAF) > 1% and imputation quality score thresholds, were used for GWAS analyses.

#### Parental smoking exposures

We explored effects of maternal and paternal smoking initiation (by using genetic variants associated with self-reported ever versus never smokers), intensity of smoking (by using genetic variants associated with self-reported number of cigarettes per day and the rs16969968 genetic variant in the *CHRNA5* gene, shown to act primarily via smoking heaviness [64, 65]). Thus, the measures we used aim to capture whether:

(i) current smoking compared to never smoking causes an effect on the outcomes of interest, and
(ii) the intensity of smoking on the outcomes of interest suggests a dose-response relationship.

This study utilised external genome-wide association studies (GWASs) carried out by the GWAS and Sequencing Consortium of Alcohol and Nicotine use (GSCAN) in 2019 and 2022 (Additional file 2: Table S1) [66, 67]. We use both 2019 and 2022 GWAS. Larger GWAS typically identify common variants with smaller effect sizes, that may be more prone to pleiotropy if the primary phenotype is imprecisely defined. Consequently, the smaller 2019 GWAS may yield estimates that are less affected by pleiotropic bias. In addition, *CHRNA5* (which is a region containing the strongest signal for smoking heaviness) [64, 65] was investigated using rs16969968 as a single instrumental variable, sourced from the above 2022 GWAS of cigarettes per day [67]. In addition, there is weak association of this variant with never and ever smoking, thus allowing stratification by these groups without introducing substantial collider bias [68]. The selected GWASs were conducted in participants of European ancestry and adjusted for principal components to minimise bias due to population stratification.

### Statistical analysis

When genetic variants are used as instrumental variables in MR, the assumptions of instrumental variables must be met, that is, the genetic variants must (i) be strongly associated with the exposure of interest and relevant to the population studied, here pregnant women (“relevance”), (ii) not share common causes with the outcome (“independence”), and (iii) not affect the outcome other than through the exposure (“exclusion-restriction”) [69].

To block the path from parental genotype through offspring genotype to the offspring outcome in an intergenerational context (which would be a violation of the “exclusion restriction” assumption), MR is robust if (i) summary outcome associations are extracted from adjusted GWAS (parental genotype adjusted for offspring genotype and if relevant, the other parent’s genotype) in a two-sample setting, or (ii) associations between parental genotype and offspring outcomes are adjusted for offspring genotype (and, where relevant, the other parent’s genotype) in a one-sample setting [6, 34, 70, 71]. There are other valid ways to conduct intergenerational one-sample MR (e.g. using maternal non-transmitted alleles) [72], which will not be used in this current study.

#### Summary level two-sample Mendelian randomisation

For the main analysis, we conducted two-sample intergenerational MR using previously derived GWAS summary statistics for each cardiometabolic risk factor (combined n for maternal, paternal and offspring GWAS = up to 564,160), which employed maternal and paternal genotype, and adjusted for the offspring’s genotype [35, 36]. These were generated using a weighted linear modelling (WLM) approach, implemented in the DONUTS R package, accounting for the correlation between the parental genotypes [73]. The WLM estimated the adjusted genetic effects (i.e. the mutually adjusted coefficients for maternal, paternal and offspring genotype, fitted jointly in the same model) as linear combinations of the unadjusted genetic effects (i.e. the coefficients for maternal, paternal and offspring genotype fitted in separate, partially overlapping samples) [70, 74, 75]. We use the shorthand “unadjusted” to refer specifically to models that are unadjusted for parental/offspring genotype, as opposed to the other covariates, including offspring age, sex, and genetic principal components, which were included in all models. We found that MR estimates changed very little when using genetic effects from a “duos” WLM (that only adjusted for offspring genotype) compared with estimates from a “trios” WLM (that adjusted for offspring genotype and the other parent’s genotype). Since the duos WLM yields smaller standard errors, we present the duos results as our primary analysis (trios results are presented in Additional file 1). GWASs were carried out on all outcomes using mother, father, and offspring genotypes. We assume that imputation quality is the same across maternal, paternal and offspring GWAS. Methods are described in detail elsewhere [35, 36]. In brief, offspring outcomes were regressed separately on maternal, paternal or offspring imputed genotype dosages, adjusting for offspring sex, age at outcome measurement (including linear and quadratic main effects and sex interaction effects, for all outcomes except birthweight), technical covariates, and the top 20 genetic principal components (calculated from maternal, paternal or offspring genotypes respectively) to adjust for population stratification. A linear mixed model using the fastGWA method [76] was used to account for cryptic relatedness. Parent-offspring pairs of European ancestry, for whom parental genotype and offspring phenotype data were available, were used. These analyses were conducted with HUNT, UKB, and ALSPAC cohorts independently and then meta-analysed using a fixed effects model implemented in METAL version 2011-03-25 [77]. Standard QC procedures were carried out including calculating the Linkage Disequilibrium Score Regression (LDSC) intercept and attenuation ratio.

**Fig. 2:**
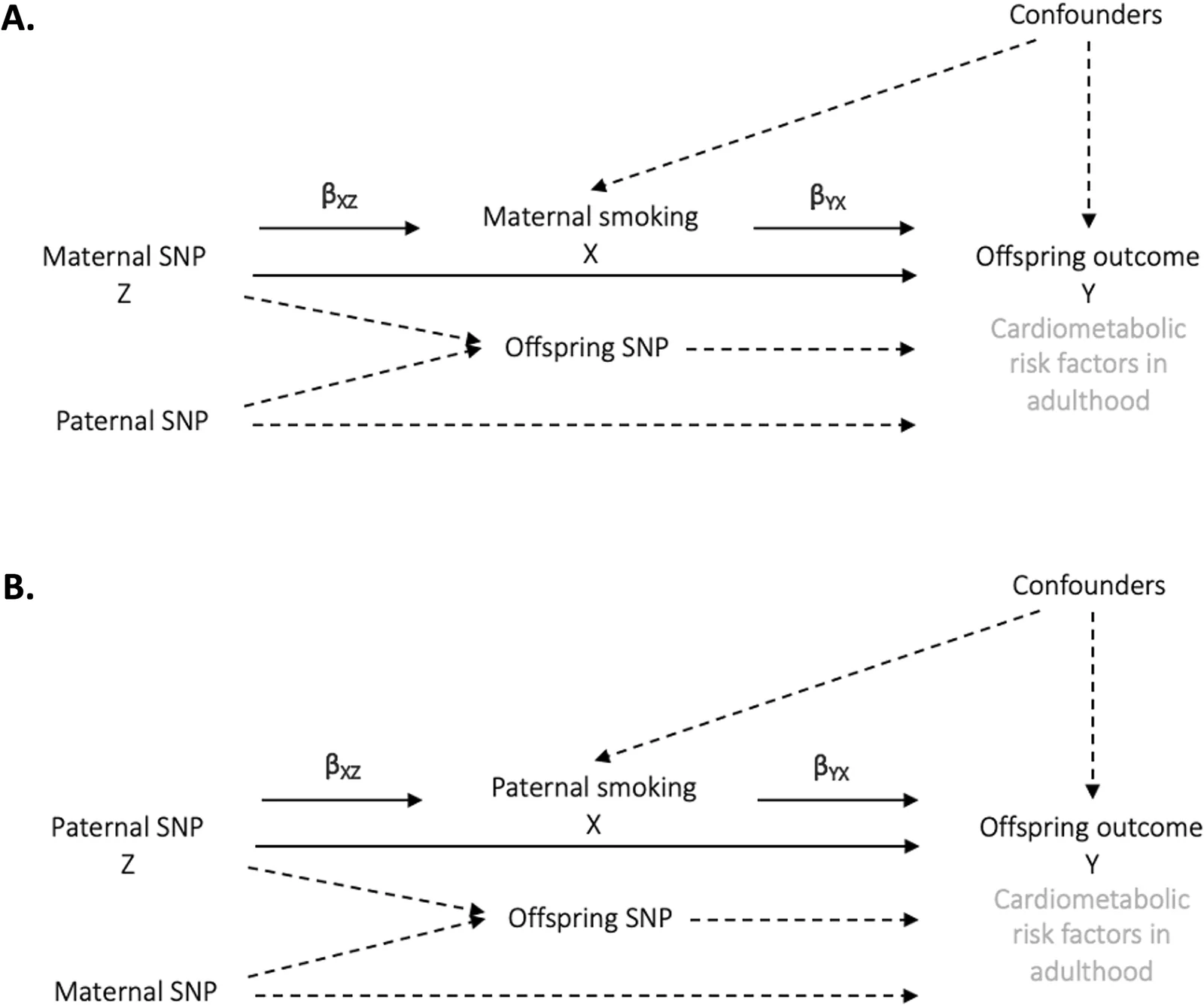
Directed acyclic graphs indicating two scenarios to explain the causal effect of parental smoking on offspring cardiometabolic risk factors in adulthood. **A**. Intergenerational MR estimating maternal smoking on offspring cardiometabolic risk factors in adulthood. The offspring and paternal SNP are adjusted for, and thus dotted paths are blocked **B**. Intergenerational MR estimating paternal smoking on offspring cardiometabolic risk factors in adulthood. The offspring and maternal SNP are adjusted for, and thus dotted paths are blocked

To estimate the causal maternal (Fig. 2A) and paternal (Fig. 2B) effects of smoking initiation and intensity on offspring outcomes, we first conducted a two-sample intergenerational MR, using the SNP-exposure estimates from the GSCAN GWAS and the SNP-outcome estimates from the adjusted maternal/paternal/offspring GWAS. We computed the inverse variance weighted (IVW) estimate, which takes a weighted mean of the variant-specific causal estimates using inverse variance weights [78]. In IVW analyses, the regression slope (ratio) is forced through a zero intercept. This assumes balanced horizontal pleiotropy. We additionally ran sensitivity analyses using MR-Egger, weighted median, and weighted mode methods [79–81]. MR-Egger evaluates the potential presence of pleiotropic effects by allowing for a non-zero intercept in the regression of the variant-outcome estimates on variant-exposure estimates [79]. It provides an unbiased estimate of the causal effect even in the presence of horizontal pleiotropy if the INstrument Strength Independent of Direct Effect (InSIDE) assumption holds [82]. The weighted median method provides an alternative estimate of the causal effect by considering the weighted median rather than the weighted mean of the instrumental variable estimates [80]. We checked for evidence of horizontal pleiotropy via Cochran’s Q test for heterogeneity, and by testing whether the MR Egger intercept differed from zero.

There is evidence that genetic variants associated with smoking also influence body composition, suggesting potential shared genetic pathways between smoking behaviours and adiposity [83, 84]. Smoking-associated genetic variants are also correlated with socioeconomic position, commonly indexed by educational attainment, indicating that these variants may capture both behavioural and socioeconomic influences [67, 85, 86]. To account for these overlapping genetic relationships, we applied multivariable MR (MVMR) in our two-sample analyses to estimate the effect of smoking initiation and smoking intensity on WHR, conditional on educational attainment and BMI. By modelling these exposures jointly, MVMR provides smoking-WHR estimates that account for these shared genetic pathways, under the multivariable instrumental variable (IV) assumptions [87, 88]. Instrument strength in the multivariable MVMR models was evaluated using conditional F-statistics, which account for the inclusion of multiple, potentially correlated exposures. These analyses were performed using the *TwoSampleMR* R package [73].

All three cohorts included in our outcome analyses (HUNT, ALSPAC and UK Biobank) contributed participants to the European-ancestry smoking GWAS (2019 and 2022) used to derive the parental smoking instruments. The extent of potential sample overlap therefore varied by GWAS year, smoking phenotype and outcome GWAS (maternal, paternal or offspring) (Additional file 2: Table S2) and reflects partial rather than complete sample non-independence. Overlap estimates are shown for BMI as a representative adult outcome GWAS; other adult outcomes were derived from the same underlying cohorts and therefore have similar overlap. Maximum potential overlap was estimated based on cohorts contributing to both the exposure and outcome GWAS, with cohort-specific overlap defined as the smaller of the cohort sample size in the exposure GWAS and the corresponding outcome GWAS. Maximum potential overlap was small for maternal and paternal outcome GWAS (<10%) but larger for offspring analyses due to the substantial contribution of UK Biobank participants to both exposure and outcome GWAS. In the presence of partial sample overlap, there is uncertainty about the direction and extent of weak instrument bias and bias due to winner’s curse. We therefore implemented overlap-robust MR using MRLap [89], to correct the IVW MR estimates for these potential biases, using linkage disequilibrium (LD) pruning with r^2^ = 0.001 and the default MRLap parameters.

Results subsequently underwent adjustment for multiple testing using Bonferroni correction for 12 outcomes (corrected significance level p = 0.004) to protect against Type 1 error [90]. Details of the input parameters are presented in Additional file 2: Table S3.

### Sensitivity analyses

#### Individual-level one-sample MR analysis

In one-sample MR, we explored effects of maternal and paternal smoking heaviness using rs16969968 in *CHRNA5*, a well-characterised functional variant associated with smoking heaviness [64, 65]. In addition, this variant has relatively weak associations with smoking initiation, facilitating stratified analyses by parental smoking status without introducing substantial collider bias [68]. These one-sample analyses represent reduced-form associations, in that offspring outcomes were regressed directly on the parental genotype without scaling by the genetic association with smoking exposure. Maternal, paternal, and offspring rs16969968 genotype were modelled assuming an additive genetic model, to estimate associations between parental rs16969968 genotype and offspring outcomes, adjusting for offspring rs16969968 genotype (and the other parent’s rs16969968 genotype). Results therefore reflect overall genetic-outcome associations, not the causal effect per unit change in smoking heaviness. We did not scale these analyses using externally estimated SNP-exposure coefficients, since smoking GWAS effect estimates are derived predominantly from general adult populations and may not precisely reflect SNP-exposure associations during pregnancy [6, 37, 91]. In addition, all cohorts included in our analyses (ALSPAC, HUNT and UK Biobank) contributed to the discovery GWAS used to obtain SNP-exposure estimates, such that scaling using externally estimated coefficients could introduce winner’s curse or sample overlap bias in this one-sample setting. These analyses were interpreted as complementary sensitivity analyses alongside the primary two-sample MR analyses. Analyses were conducted in up to 17,484 mother-father-offspring trios from HUNT, UKB and ALSPAC.

We fitted a linear mixed model implemented in the Genome-wide Complex Trait Analysis (GCTA) software package [92] using a genetic relatedness matrix (GRM) constructed from the offspring genotypes to account for relatedness and population stratification. rs16969968 in *CHRNA5* and any SNPs within ±1 Mb of the locus (to account for LD) were excluded from GRM construction [6]. All analyses were adjusted for offspring age at measurement and sex. The GRM mitigated the need to adjust for principal components (PCs). Parent-offspring pairs of European ancestry, for whom parental genotype and offspring phenotype data were available, were used. We implemented a complete-case approach, excluding individuals with missing values from the analysis. These analyses were conducted in HUNT, UKB, and ALSPAC, and then meta-analysed. A fixed-effects meta-analysis of regression coefficients from the three cohorts was conducted using Stata version 16 [93].

#### Stratified analysis

If our genetic instruments for smoking heaviness (including rs16969968 in the *CHRNA5* gene) are valid, we would expect these variants to only indicate a causal effect when the mother or father is a smoker. If a causal effect is detected in parental never-smokers, this implies that the *CHRNA5* gene is influencing the offspring outcome via mechanisms other than smoking heaviness (i.e. horizontal pleiotropy). We therefore repeated the *CHRNA5* gene MR analyses, stratifying the sample to parental ever-smokers and never-smokers [64]. For two-sample analyses, we only had a sufficiently large sample to run stratified analyses for birthweight (because the WLM relies on LDSC [94], which has stringent sample size requirements), whereas we ran stratified one-sample analyses for adult outcomes too.

## Results

The GWAS meta-analyses used for the SNP-outcome association in the two-sample MR included between 14,481 (for HbA1c with paternal genotype) and 511,253 (for DBP with offspring genotype) participants (Additional file 2: Table S4) [35, 36].

**Fig. 3:**
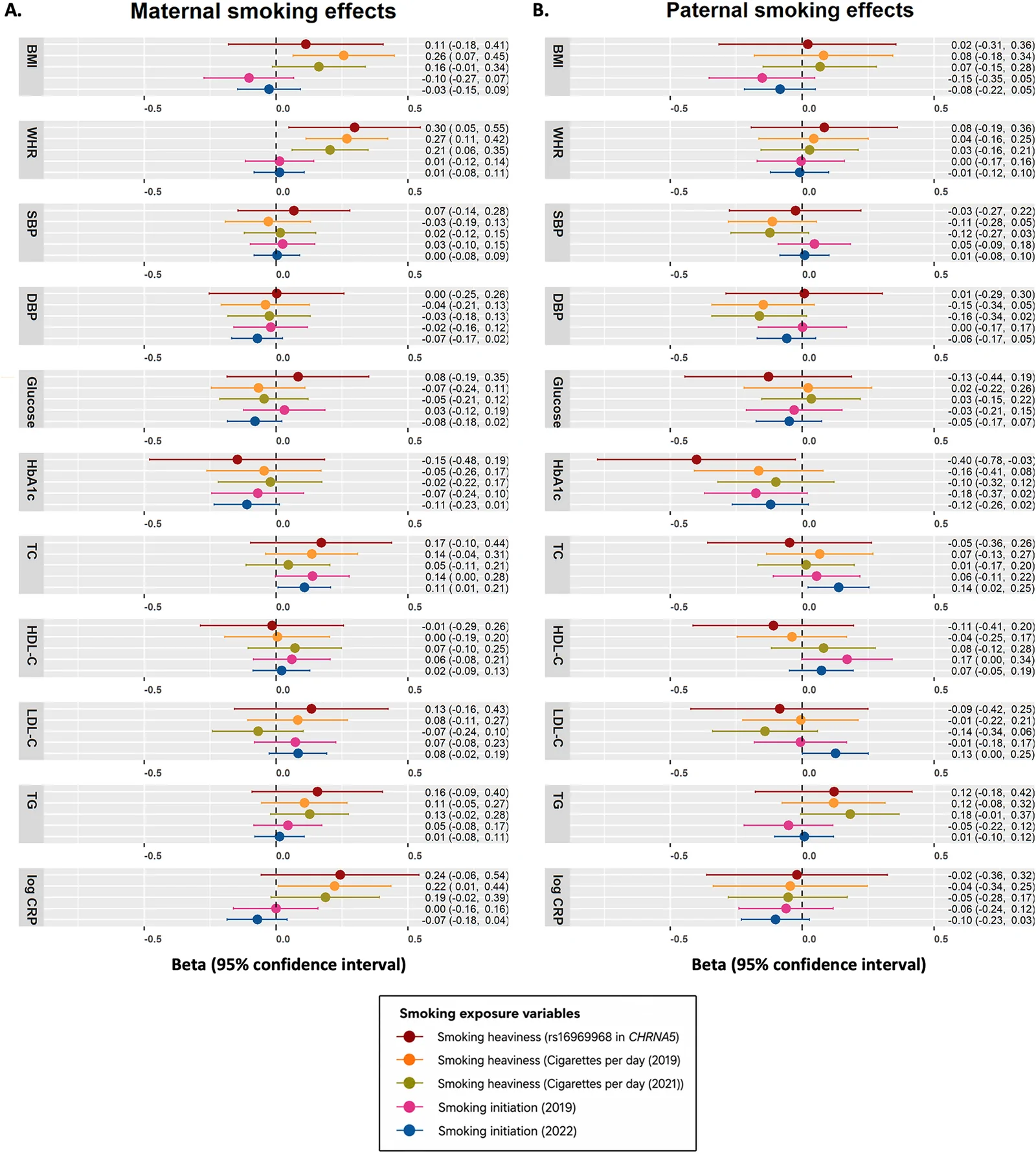
Two-sample Mendelian randomization (MR) for maternal and paternal smoking heaviness and initiation onto 11 long-term cardiometabolic outcomes in offspring. Parental genotype was adjusted for offspring genotype (duos WLM). **A**. Maternal smoking on offspring cardiometabolic risk factors in adulthood. **B**. Paternal smoking on offspring cardiometabolic risk factors in adulthood. MR estimates for the effect of smoking heaviness can be interpreted as the average change in the outcome, in standard deviation (SD) units, per one SD increase in parental cigarettes smoked per day. MR estimates for the effect of smoking initiation can be interpreted as the average change in the outcome, in SD units, per doubling in the odds of smoking initiation

We found strong evidence of an average change in adult offspring WHR per one standard deviation (SD) increase in maternal cigarettes smoked per day of 0.25 SD (95% confidence interval (CI): 0.13, 0.38) using GWAS carried out in 2019, 0.19 SD (95% CI: 0.08, 0.31) using the GWAS carried out in 2022, and 0.27 SD (95% CI: 0.07, 0.48) using *CHRNA5* rs16969968 (Fig. 3A, Additional file 2: Table S5). Corresponding paternal estimates provided little evidence of an effect on offspring WHR, with an average change per one SD increase in paternal cigarettes smoked per day of −0.06 SD (95% CI: −0.23, 0.12) using the 2019 GWAS and −0.04 SD (95% CI: −0.20, 0.11) using the 2022 GWAS, and −0.02 SD (95% CI: −0.25, 0.22) using *CHRNA5* rs16969968 (Fig. 3B, Additional file 2: Table S6). The estimates presented here all derive from unstratified analyses; stratified analyses are presented later. There was weaker evidence that greater maternal smoking heaviness had an effect on higher BMI and CRP in adult offspring. For BMI, the average change in offspring BMI per one SD increase in maternal cigarettes smoked per day was 0.23 SD (95% CI: 0.07, 0.39) using the 2019 GWAS instrument, 0.14 SD (95% CI: 0.00, 0.29) using the 2022 GWAS instrument, and 0.11 SD (95% CI: −0.13, 0.35) using *CHRNA5* rs16969968. For CRP, the average change in offspring CRP per one SD increase in maternal cigarettes smoked per day was 0.24 SD (95% CI: 0.08, 0.40) using the 2019 GWAS instrument, 0.20 SD (95% CI: 0.04, 0.36) using the 2022 GWAS instrument, and 0.25 SD (95% CI: 0.01, 0.49) using *CHRNA5* rs16969968. Corresponding paternal estimates showed little evidence of effects on BMI and CRP (Fig. 3B, Additional file 2: Table S6). Most other paternal estimates were imprecise and compatible with the null, with only a small number suggesting possible effects for other cardiometabolic traits (e.g. HbA1c and blood pressure); these did not withstand correction for multiple testing. Full estimates are provided in Additional file 2: Table S6. In the analyses using the trios WLM for the outcome GWAS (in which the parental genetic effects were adjusted for the other parent’s genotype as well as the offspring’s genotype), results were similar, although the confidence intervals were wider, likely due to the lower statistical power of the trios WLM versus the duos WLM (Additional file 1: Figure S2).

Point estimates from the two-sample weighted median, weighted mode and MR-Egger methods were similar to IVW estimates and evidence remained robust for the maternal effect for smoking heaviness on offspring WHR in later life (Additional file 2: Table S5). In addition, Cochran’s Q test provided little evidence for between-SNP heterogeneity, and there was not strong evidence that the MR Egger intercept differed from zero (Additional file 2: Table S7). This suggests little evidence that horizontal pleiotropy is driving these results. After correction for multiple testing using a Bonferroni test, evidence of an effect remained only for maternal smoking heaviness on increased WHR and BMI (p = 0.004). Findings for adult offspring outcomes using the unadjusted GWAS sensitivity analyses are presented in Additional file 2: Table S8 for maternal exposures and Additional file 2: Table S9 for paternal exposures.

MVMR analyses indicated that there remained strong evidence for the effect of maternal smoking heaviness on offspring WHR in later life after accounting for BMI as well as educational attainment (Additional file 2: Table S10). There was little evidence of an effect of either educational attainment or BMI on WHR in offspring after adjustment for any maternal smoking indicator. Comparison of the conditional F-statistics showed that the instruments for BMI and educational attainment were substantially stronger than those for the smoking exposures (Additional file 2: Table S10). The strength of the BMI and educational attainment instruments was largely preserved when conditioned on smoking, indicating that these estimates were not affected by SNP non-overlap. In contrast, conditioning led to considerable weakening of the smoking instruments, consistent with the shared genetic architecture between smoking behaviour and BMI. Accordingly, estimates for smoking heaviness should be interpreted with some caution; however, the persistence of an association with offspring WHR after adjustment for BMI suggests that this effect estimate is not solely attributable to shared genetic pathways and may in fact be attenuated by weak-instrument bias.

Estimates obtained using MRLap were very similar to IVW estimates across outcomes, including the positive association between maternal smoking heaviness and offspring WHR. Paternal estimates likewise remained centred around the null (Additional file 2: Table S11), suggesting that partial sample overlap is unlikely to materially influence the causal estimates.

### Sensitivity analyses

#### Individual-level one-sample MR analysis

**Fig. 4:**
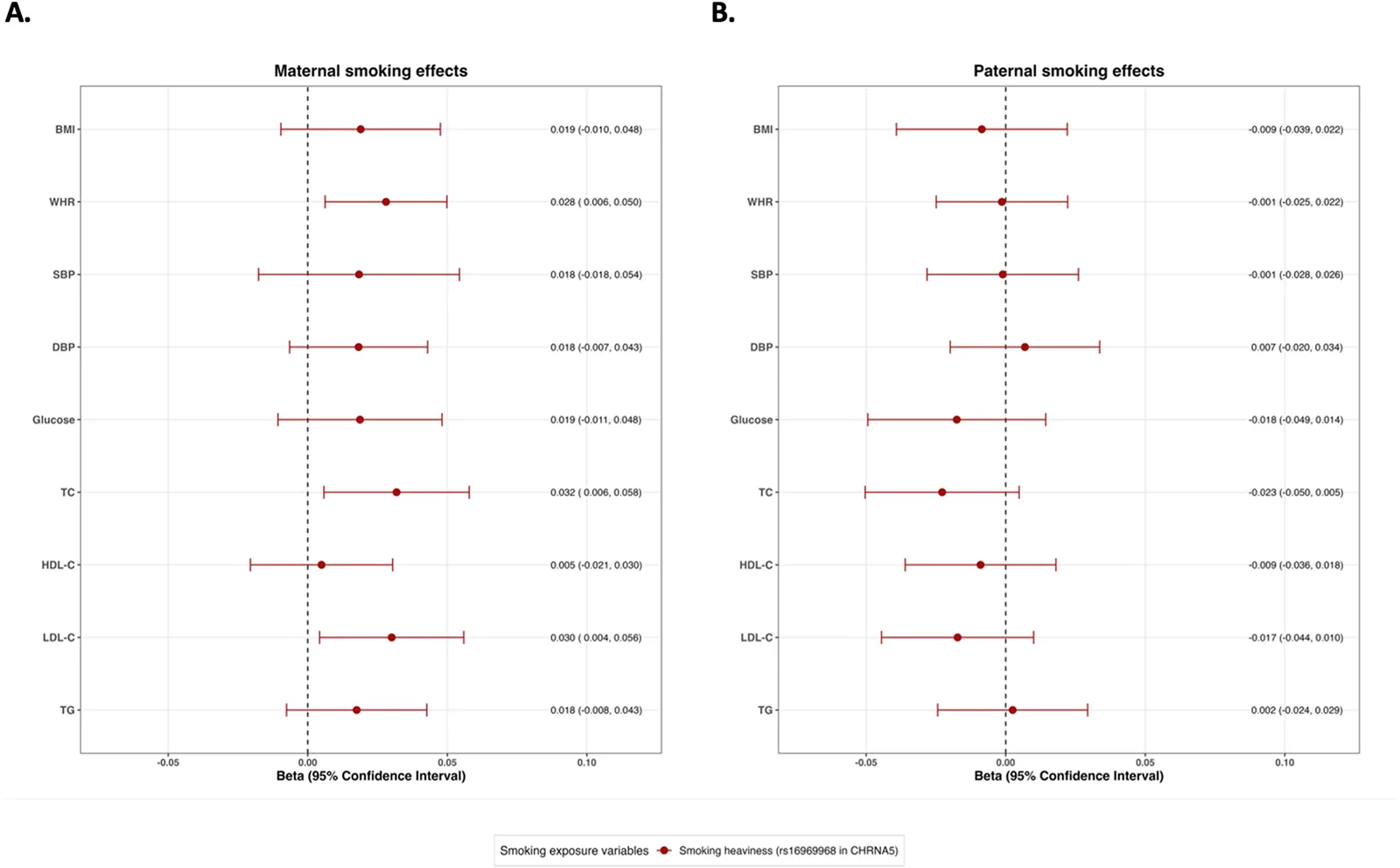
One-sample Mendelian randomization (MR) for smoking heaviness onto 9 long term cardiometabolic outcomes in offspring. **A**. Maternal smoking on offspring cardiometabolic risk factors in adulthood. **B**. Paternal smoking on offspring cardiometabolic risk factors in adulthood. Estimates represent the average change in offspring cardiometabolic outcomes per copy of the *CHRNA5* risk allele, expressed in standard deviation (SD) units of each outcome. These estimates reflect associations between the parental genetic instruments and offspring outcomes, rather than causal effects per unit change in smoking heaviness

The individual-level one-sample analysis comprised between 16,942 and 17,484 mother-father-offspring trios (Additional file 2: Table S12). These one-sample analyses represent reduced-form associations between parental genetic scores and offspring outcomes and are included as complementary sensitivity analyses alongside the primary two-sample MR results. One-sample MR in the total population indicated that maternal smoking heaviness instrumented by rs16969968 at *CHRNA5* showed evidence consistent with a positive association with offspring WHR in later life, with weaker evidence for effects on total cholesterol and LDL-cholesterol (Fig. 4A, Additional file 2: Table S13). Paternal smoking heaviness showed little evidence of an association with offspring WHR and other cardiometabolic traits in one-sample MR (Fig. 4B, Additional file 2: Table S14). Cohort-specific results are presented in Additional file 2: Tables S15–S17.

#### Stratified analysis

**Fig. 5:**
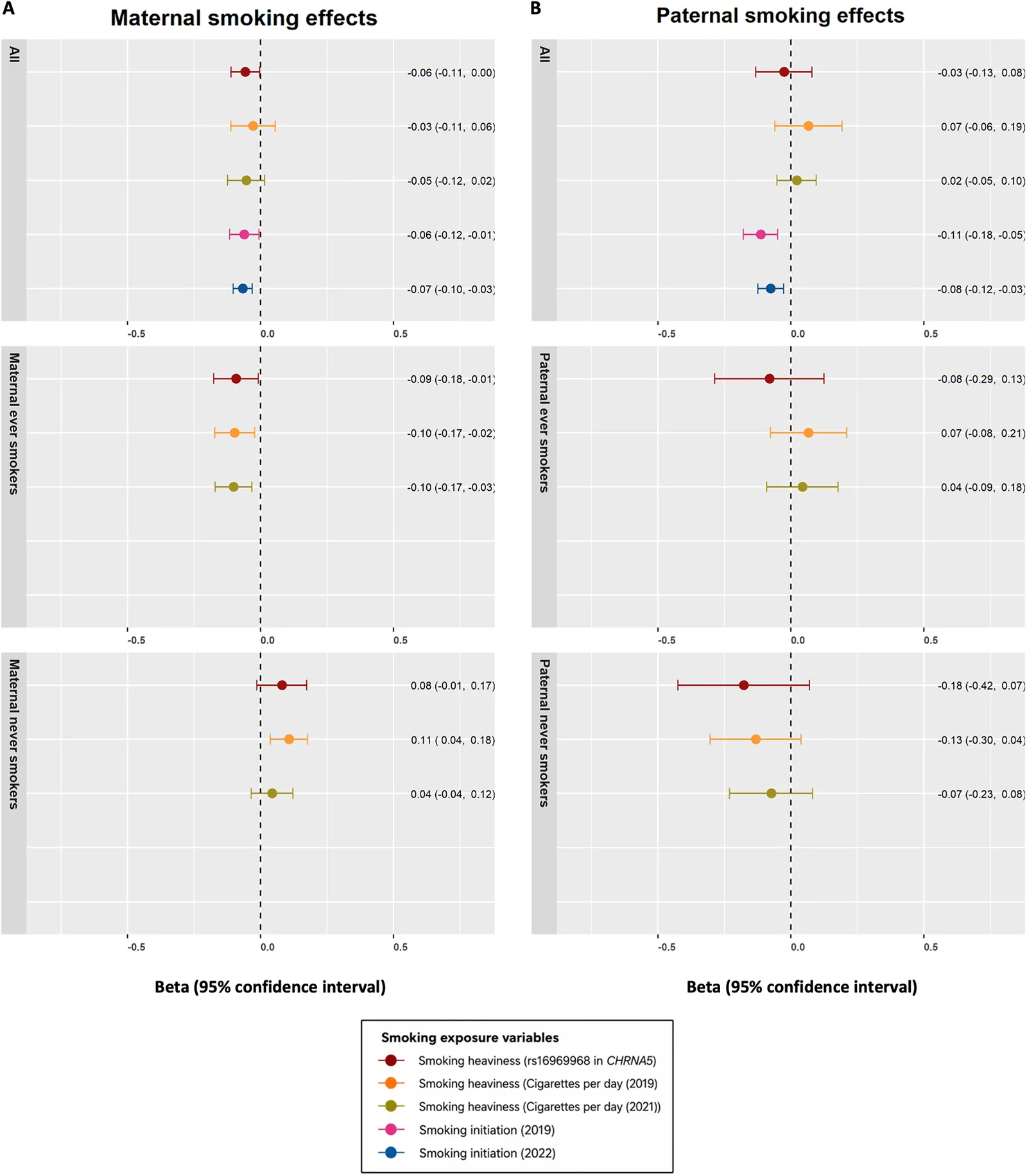
Two-sample Mendelian randomization (MR) for maternal and paternal smoking heaviness and initiation onto birthweight in offspring, stratified by parental smoking status, using unadjusted (marginal) genetic effects. **A**. Maternal smoking exposures on birthweight. **B**. Paternal smoking exposures on birthweight. MR estimates for the effect of smoking heaviness can be interpreted as the average change in the outcome, in standard deviation (SD) units, per one SD greater parental cigarettes smoked per day. MR estimates for the effect of smoking initiation can be interpreted as the average change in the outcome, in SD units, per doubling in the odds of smoking initiation

For GWAS of birthweight stratified by parental smoking status, analysis comprised between 10,719 and 136,933 participants in maternal ever smokers, 8,094 and 104,002 participants in maternal never smokers, 9,425 and 27,726 participants in paternal ever smokers, and 5,367 and 18,718 participants in paternal never smokers (Additional file 2: Table S18). In analyses employing the unadjusted GWAS (i.e. using genetic effects that were not adjusted for the offspring’s or other parent’s genotype), in the unstratified sample (including parental never and ever smokers) we found suggestive evidence that maternal and paternal smoking initiation caused lower birthweight (Fig. 5; Additional file 2: Table S19). We also found some evidence that greater maternal smoking heaviness caused lower offspring birthweight. In the maternal ever smoker group, these effects were noticeably stronger. In the maternal never smoker group the effects of maternal smoking heaviness flipped sign and became weakly positive. Corresponding paternal smoking heaviness estimates in both unstratified and stratified birthweight analyses were centred around the null (Fig. 5, Additional file 2: Table S19). Taken together, these observations support our use of birthweight as a positive control outcome. In the analyses using the adjusted GWAS, the direction of effect was often consistent with analyses using the unadjusted GWAS, but the adjusted GWAS had lower statistical power, resulting in weaker evidence (Additional file 1: Figure S3, Additional file 1: Figure S4).

**Fig. 6:**
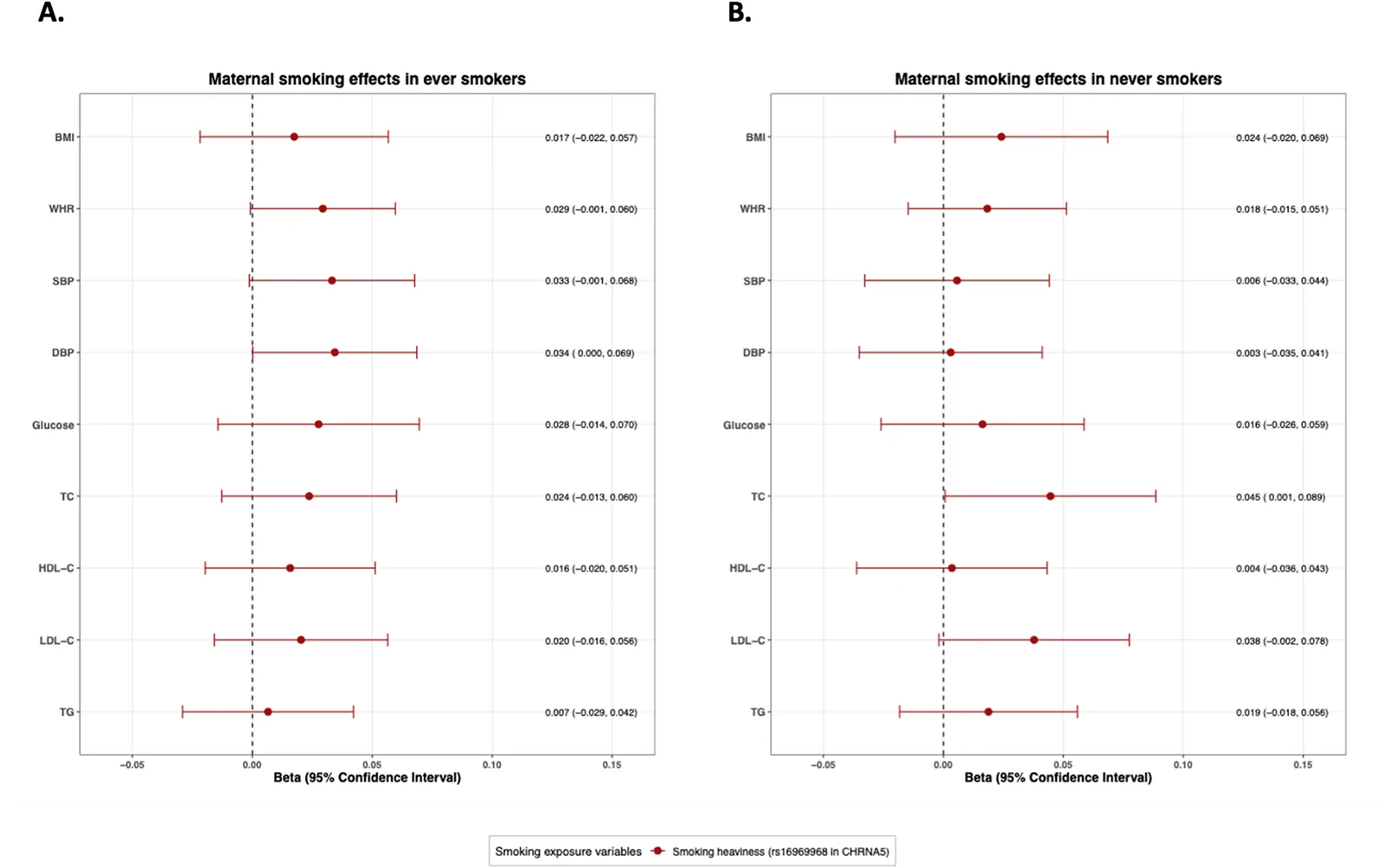
One-sample Mendelian randomization (MR) for maternal smoking heaviness onto 9 long-term cardiometabolic outcomes in offspring in maternal ever and never smokers. **A**. Maternal smoking on offspring cardiometabolic risk factors in adulthood in maternal ever smoking group. **B**. Maternal smoking on offspring cardiometabolic risk factors in adulthood in maternal never smokers. Estimates represent the average change in offspring cardiometabolic outcomes per copy of the *CHRNA5* risk allele, expressed in standard deviation (SD) units of each outcome. These estimates reflect associations between the parental genetic instruments and offspring outcomes, rather than causal effects per unit change in smoking heaviness

The one-sample MR stratified smoking samples comprised between 8,814 and 9,065 participants in maternal ever smokers, 7,357 and 7,624 in maternal never smokers, 12,004 and 12,339 in paternal ever smokers, and 4,445 and 4,623 in paternal never smokers, with numbers differing across outcomes (Additional file 2: Table S12). Whilst the sample size was smaller, and thus power was low, the magnitude of effect became slightly stronger in ever smokers for the effects of maternal smoking heaviness instrumented by rs16969968 in *CHRNA5* on WHR (Fig. 6A, Additional file 2: Table S13). There was additionally some evidence of an association of maternal smoking heaviness with DBP and SBP in ever smokers. There was weak evidence of an association of maternal smoking heaviness with TC and LDL-cholesterol in never smokers, although this could have been attributable to Type 1 error (Fig. 6B, Additional file 2: Table S13). In paternal ever and never smokers, effect estimates were generally close to the null across cardiometabolic outcomes (Additional file 2: Table S14). An association between paternal smoking heaviness and triglycerides was observed in paternal never smokers, arguing against a smoking-specific causal interpretation, since no effect of smoking heaviness would be expected among never smokers. As with all stratified analyses, the potential for collider bias should be considered when interpreting these results.

## Discussion

Employing intergenerational MR analyses across three large cohorts, we found robust evidence supporting a causal effect of greater maternal smoking heaviness (but not initiation) on higher WHR in adult offspring. We also found suggestive evidence that greater maternal smoking heaviness causes greater offspring adult BMI and circulating CRP. We did not find robust evidence of maternal smoking effects on other cardiometabolic risk factors or of paternal smoking effects on any of the outcomes investigated. The MR models in this study cannot distinguish whether the estimated effects reflect maternal or paternal smoking before conception, during pregnancy, or after birth. They are therefore not specific to exposure in pregnancy, and we cannot determine from these results whether the observed effect of maternal smoking on adult offspring WHR is driven by preconception, intrauterine or postnatal mechanisms. Importantly, stronger maternal effects could reflect greater postnatal environmental tobacco smoke exposure, given that mothers have historically spent more time in close contact with children during early life. In this scenario, a larger maternal effect would not necessarily imply intrauterine programming but could instead reflect higher cumulative childhood exposure to second-hand smoke. Although the weaker paternal effects argue against a purely postnatal explanation, unmeasured differences in parental caregiving, contact, or social expectations may limit the validity of paternal smoking as a negative control for postnatal exposure. Thus, our findings may reflect true effects that are similar across exposure to maternal smoking at different life stages, or effects operating across multiple exposure windows or varying across sensitive periods that our design cannot disentangle. If the assumptions underpinning our analyses hold, this study is consistent with previous non-genetic epidemiological findings, which identify an association between maternal smoking and higher central adiposity in offspring, that persists into adulthood [14, 15]. Although there was some indication of an effect of maternal smoking on BMI and circulating CRP in the two-sample analyses, the BMI effect was weaker than that observed for WHR and did not replicate in the one-sample MR. CRP was not available for one-sample analysis. Overall, this pattern may suggest that any influence of maternal smoking heaviness relates specifically to the fat mass component of BMI.

Several potential mechanisms for the effect of maternal smoking on offspring adiposity have been proposed. In animal studies, maternal nicotine exposure has been associated with alterations in adipose tissue and glucose metabolism [95, 96]. Although reduced fetal growth has been suggested as a DOHaD-related mechanism [97, 98], evidence from MR studies provides limited support for broad intrauterine programming effects [72], and such interpretations should therefore be made cautiously. Observational studies also show that associations between parental smoking and offspring overweight are often independent of fetal or postnatal growth and birthweight, which further limits support for a fetal growth–mediated explanation [13]. Furthermore, sibling studies have reported that the association between parental smoking and offspring obesity/overweight in childhood and adulthood attenuates when assessed within siblings [24, 25]. Such attenuation could indicate that familial confounding is an important driver of these associations, although the sibling estimates typically have wide confidence intervals which would also be consistent with meaningful causal effects. It is also difficult to directly compare effect sizes from our MR analyses with those from previous sibling studies, due to differences in the definition and measurement units of the smoking exposures and in estimands obtained across methods.

The limited evidence we observed for an effect of paternal smoking on WHR in adult offspring may be considered in the context of previous maternal and paternal comparison studies [26, 99]. Such studies are often interpreted under the assumption that, if an intrauterine effect exists, the association of maternal smoking with offspring outcomes should be stronger than the equivalent paternal association [100]. Earlier observational investigations and meta-analyses appeared consistent with this assumption, reporting more adverse maternal relative to paternal associations of parental smoking with childhood overweight and obesity [26, 99, 101]. However, maternal-paternal comparisons are generally more informative for pregnancy outcomes, which occur during the exposure window itself, than for long-term offspring outcomes [27], where postnatal environmental and familial influences can accumulate and obscure intrauterine effects. Consistent with this, a more recent individual participant data meta-analysis including 229,158 families [28] reported maternal and paternal associations of comparable magnitude for childhood overweight, indicating that earlier maternal-paternal differences may have reflected residual confounding rather than true intrauterine effects. These limitations underscore the value of approaches such as MR in differentiating putative intrauterine causal effects from shared familial confounding when interrogating long-term offspring phenotypes.

Single-trait assortative mating, or nonrandom matching between spouses, occurs when partners are selected on the basis of a single phenotype [102]. For instance, if individuals who smoke are more likely to select partners who also smoke, the resulting enrichment of smoking-concordant couples could bias associations in our study. In using a WLM to estimate parental genetic effects, we assume that the same phenotype was measured in the samples of mothers, fathers, and offspring, and that cross-locus genetic correlations due to assortative mating are absent. In addition, within a family context, it is difficult to fully unpick the effects of individual behaviour. Whilst it has been shown that the DNA methylation signature associated with passive smoke exposure is much less pronounced than that of own smoking [103], passive smoking or offspring’s own smoking behaviour may still have an impact on findings. Therefore, the effects that we found may encompass (i) maternal exposure to second-hand smoke from the partner during pregnancy, due to unmodelled assortative mating effects, (ii) postnatal exposure to second-hand smoke during the offspring’s childhood, and (iii) offspring smoking in adolescence and early adulthood.

Another consideration is that the smoking genetic instruments were derived from large sex-combined GWAS meta-analyses. Although smoking behaviour differs phenotypically between males and females, available evidence suggests largely shared genetic architecture. For example, genetic correlations between males and females for smoking-related traits in UK Biobank have been reported as 0.96 for current tobacco smoking and 0.89 for smoking status [104]. Large sex-stratified GWAS of comparable size are not currently available. However, a high genome-wide genetic correlation does not guarantee that individual SNPs have equivalent predictive performance across sexes, and modest sex-specific differences in SNP effects cannot be excluded.

There are several strengths of this study. For the main analysis, we took advantage of novel statistical methods [34, 73, 75, 105] recently developed to conduct intergenerational two-sample MR, by estimating the effects of parental genotype on offspring outcomes in adulthood, adjusting for the offspring’s own genotype and (when necessary) the other parent’s genotype. This approach removed the need to analyse a subset of individuals with complete maternal, paternal, and offspring genotype data available (as we have done for the one-sample MR analyses). In addition, we applied linear mixed model association methods to data from large population-based cohorts that comprised related individuals: HUNT, UKB and ALSPAC, increasing our available sample size considerably. We adjusted for the offspring’s and other parent’s genotype, which is crucial to preserve the key exclusion restriction assumption of MR [30]. Importantly, we included birthweight as a positive control outcome, demonstrating that our method is able to detect the expected causal effect of maternal smoking on offspring birthweight. In particular, our MR results indicated an effect of both maternal and paternal smoking initiation on offspring birthweight. However, the presence of a paternal effect raises the possibility that the smoking initiation instrument may be picking up effects of other exposures besides smoking, via horizontal pleiotropy. We were also able to stratify MR analyses by parental smoking status; if parental smoking heaviness exhibits a putative causal effect in parental never smokers, this indicates our method is biased. We found some evidence that heavier maternal smoking causes lower offspring birthweight in the unstratified sample, but these effects were stronger in ever smokers.

An important limitation of this study is that despite relatively large sample sizes, low power was an issue, particularly within some of the stratified sensitivity analyses. These analyses should therefore be interpreted with appropriate caution. Moreover, smoking initiation is an example of a complex behavioural exposure where pleiotropy may be operating [106, 107]. Smoking initiation has been shown to associate with risk-taking behaviours, personality traits, and externalising disorders [85, 86]. To strengthen this study, we explored several indicators for smoking and conducted multiple sensitivity analyses. The relationship between smoking and body composition is particularly complex [83, 84]. Suggestions of a common biological basis for nicotine addiction and obesity have been proposed, with higher adiposity influencing smoking behaviour [84, 108]. This link identified between obesity and smoking behaviour may have implications for weight control and smoking prevention strategies [84]. We identified several body composition measures which exhibit high genetic correlation with smoking indicators in publicly available GWAS data [67]. Our MVMR analyses suggest that smoking initiation may be an unreliable exposure for MR analyses. In addition, a potential source of bias could arise from gene-sex interactions in the two-sample WLM analyses, if residual sex differences in SNP-outcome effects remain even after adjusting for offspring sex. This consideration is particularly relevant for WHR, which has well-established sex-specific genetic architecture [109], and in theory this could contribute to the stronger maternal MR estimate observed for this trait. However, since we observed similar maternal effects on BMI and CRP, which are less sexually dimorphic genetically, this is unlikely to be a major source of bias for our MR analyses. While all our outcome GWAS adjusted for offspring sex, they did not explicitly model gene-sex interactions, so some residual bias cannot be ruled out. Despite this, previous findings suggest that maternal and paternal BMI and glucose traits both exhibit null MR effects on offspring cardiometabolic risk factors in this data set [35, 36]. Such bias is therefore unlikely to fully explain these patterns of associations. Offspring age at cardiometabolic assessment differed across cohorts. In ALSPAC, outcomes were measured at age 24 years (early adulthood), whereas participants in HUNT and UK Biobank were assessed in mid-to-later adulthood. If adverse cardiometabolic effects of parental smoking accumulate or become more pronounced with age, effect estimates at age 24 may be smaller than those observed at older ages. Inclusion of ALSPAC would therefore be expected to attenuate pooled estimates. We also excluded any genetic variants from the two-sample MR analyses if they had strong evidence for between-variant heterogeneity (Cochran’s Q test p < 0.001) in any of the outcome (parental or offspring) GWAS. It is therefore unlikely that any between-cohort heterogeneity in the outcome GWAS meaningfully affected our MR results. Finally, our study included only participants of White European ancestry from high-income countries, therefore our results may not be generalisable to other populations.

## Conclusions

In conclusion, our study provides evidence supporting a causal effect of heavier maternal smoking on greater WHR in adult offspring, with more tentative indications of effects on offspring BMI and circulating CRP. We did not find robust evidence for maternal smoking effects on other cardiometabolic risk factors, or for effects of paternal smoking on any of the cardiometabolic factors examined. Triangulation with complementary study designs will be important for strengthening causal inference, and our findings, which are compatible with causal effects of maternal smoking on central adiposity in adult offspring, reinforce the public-health rationale for promoting smoking cessation prior to pregnancy.

## Supplementary information

Supplementary Material 1: Additional file 1: Supplementary information, Figures S1–S4.

Supplementary Material 2: Additional file 2: Tables S1–S19.

## Data Availability

Data access for the Avon Longitudinal Study of Parents and Children (ALSPAC) operates via a managed open access system. Approved proposals are reviewed by the ALSPAC Executive Committee. Full details are provided in the ALSPAC Data Management Plan (https://www.bristol.ac.uk/alspac/researchers/data-access/documents/alspac-data-management-plan.pdf). To request access to data from the Trøndelag Health Study (HUNT), researchers affiliated with Norwegian research institutes can apply for the use of HUNT data and biological samples, subject to approval by the Regional Committee for Medical and Health Research Ethics. Researchers from other countries may also apply if collaborating with a Norwegian Principal Investigator. All applications are reviewed by the HUNT Data Access Committee, and successful applicants are required to enter into data access and/or material transfer agreements. Detailed information on the application process, ethical requirements, and available datasets can be found on the HUNT website (https://www.ntnu.edu/hunt/data). Access to UK Biobank (UKB) data is obtained through a standard application process. Researchers register with UKB, submit a study proposal, and obtain approval from the UKB Access Management Team. Approved applicants receive access to de-identified data under UKB’s terms of use. Further information is available on the UK Biobank website (https://www.ukbiobank.ac.uk/enable-your-research/apply-for-access). None of the material has been published or is under consideration for publication elsewhere.

## Abbreviations

ALSPAC: Avon Longitudinal Study of Parents and Children
BMI: Body mass index
CHRNA5: Cholinergic receptor nicotinic alpha 5 subunit gene
CI: Confidence interval
CRP: C-reactive protein
CVD: Cardiovascular disease
DBP: Diastolic blood pressure
DOHaD: Developmental Origins of Health and Disease
DNA: Deoxyribonucleic acid
GCTA: Genome-wide Complex Trait Analysis
GRM: Genetic relatedness matrix
GSCAN: GWAS and Sequencing Consortium of Alcohol and Nicotine use
GWAS: Genome-wide association study
HbA1c: Glycated haemoglobin
HDL-C: High-density lipoprotein cholesterol
HRC: Haplotype Reference Consortium
HUNT: Trøndelag Health Study
IV: Instrumental variable
IVW: Inverse variance weighted
LD: Linkage disequilibrium
LDL-C: Low-density lipoprotein cholesterol
LDSC: Linkage Disequilibrium Score Regression
MAF: Minor allele frequency
MBRN: Medical Birth Registry of Norway
MR: Mendelian randomization
MR-Egger: Mendelian randomization-Egger
MVMR: Multivariable Mendelian randomization
PC: Principal component
QC: Quality control
REDCap: Research Electronic Data Capture
SBP: Systolic blood pressure
SD: Standard deviation
SNP: Single nucleotide polymorphism
STROBE-MR: Strengthening the Reporting of Observational Studies in Epidemiology using Mendelian Randomization
UKB: UK Biobank
WHR: Waist-hip-ratio
WLM: Weighted linear modelling
